# Register-based data of psychosocial working conditions and occupational groups as predictors of disability pension due to musculoskeletal diagnoses: a prospective cohort study of 24 543 Swedish twins

**DOI:** 10.1186/1471-2474-14-268

**Published:** 2013-09-16

**Authors:** Annina Ropponen, Åsa Samuelsson, Kristina Alexanderson, Pia Svedberg

**Affiliations:** 1 Finnish Institute of Occupational Health, Helsinki, Finland; 2 Division of Insurance Medicine, Department of Clinical Neuroscience, Karolinska Institutet, Stockholm, Sweden

**Keywords:** Sick leave, Disability pension, Psychosocial working conditions, Occupational group, Musculoskeletal disorder, Twin

## Abstract

**Background:**

Occupations and psychosocial working conditions have rarely been investigated as predictors of disability pension in population-based samples. This study investigated how occupational groups and psychosocial working conditions are associated with future disability pension due to musculoskeletal diagnoses, accounting for familial factors in the associations.

**Methods:**

A sample of 24 543 same-sex Swedish twin individuals was followed from 1993 to 2008 using nationwide registries. Baseline data on occupations were categorized into eight sector-defined occupational groups. These were further used to reflect psychosocial working conditions by applying the job strain scores of a Job Exposure Matrix. Cox proportional hazard ratios (HR) were estimated.

**Results:**

During the 12-year (average) follow-up, 7% of the sample was granted disability pension due to musculoskeletal diagnoses. Workers in health care and social work; agriculture, forestry and fishing; transportation; production and mining; and the service and military work sectors were two to three times more likely to receive a disability pension than those in the administration and management sector. Each single unit decrease in job demands and each single unit increase in job control and social support significantly predicted disability pension. Individuals with high work strain or an active job had a lower hazard ratio of disability pension, whereas a passive job predicted a significantly higher hazard ratio. Accounting for familial confounding did not alter these results.

**Conclusion:**

Occupational groups and psychosocial working conditions seem to be independent of familial confounding, and hence represent risk factors for disability pension due to musculoskeletal diagnoses. This means that preventive measures in these sector-defined occupational groups and specific psychosocial working conditions might prevent disability pension due to musculoskeletal diagnoses.

## Background

Disability pension (DP) due to musculoskeletal diagnoses (MSD) is currently one of the most common causes for early exit from the labour market [1,2]. Investigations of work-related risk factors for such DP have revealed that socioeconomic status, physical workload, and psychosocial aspects influence the risk of DP [3-8]. The few studies that have examined psychosocial working conditions have detected associations between low job control [9,10], high job strain [11-14] and DP. Furthermore, psychosocial working conditions have shown to mediate the associations between the type of occupation and DP [5]. The association between job strain and DP due to MSD [14] has been previously investigated, as has the association with low job control [10], but the associations between psychosocial working conditions and DP due to MSD still need to be clarified.

Factors other than working conditions are also associated with the risk of DP, for example, type of occupational group. Previous studies examining the role of occupational groups have focused on socioeconomic status and revealed clear associations between occupational socioeconomic status and DP. For example, white-collar workers seem to be at a lower risk, whereas manual workers are at a higher risk of DP due to MSD [5,7,8]. However, there is a lack of studies with more detailed information on socioeconomic status, or a classification of occupations in the population-based samples. Two studies exist that seem to point to differences in the risk for DP due to MSD between different occupational groups [4,15].

Another important aspect when studying the risk factors for DP due to MSD is the moderate genetic component, which accounts for 35–37% of the variation in such DP [16,17]. Individual-related environmental effects, which are mainly exposures or circumstances encountered in adulthood, such as education, occupation, or psychosocial working conditions, account for the remaining 63–65% of variance of DP due to MSD. The clear influence of these environmental factors implies that work-related factors may be modifiable risk factors for DP due to MSD, and could potentially represent a basis for interventions or other preventative actions. Therefore, differentiating between environment and heredity in these studies is important and can be achieved using twin data which allows control of familial confounding (genetics and family environment) by identifying twin pairs who are discordant for DP and for the risk factors of interest. The co-twin control design is a strong method of case–control design, as same-sexed twins are by definition the same age and sex. In the co-twin control setting, twins with different working conditions or occupational groups provide an opportunity to study the underlying causal pathways behind these associations. If familial factors are of importance, then no association should be present between work-related factors and DP within the discordant twin pairs. However, if factors specific to each individual are more important, then associations found in discordant twin pairs will be of a similar magnitude to those in the analyses between all individuals. To the best of our knowledge, no previous study has evaluated the associations between occupation and psychosocial working conditions with DP due to MSD to determine the impact of familial factors.

Furthermore, despite the relatively abundant research on occupational groups, work-related factors and risk of DP due to MSD, most previous studies have used self-reported data for the determination of socioeconomic status, occupational groups, or work loading [7,8,10,13,18,19]. A few studies have included some register data related to, for example, socioeconomic status [7,20] or sickness absence [21], but investigators have seldom had access to register data regarding more comprehensive work-related factors [14]. Large data, preferably from high quality registers, on exposures as well as on DP might provide new insights into the interplay of work-related factors with DP. Swedish nationwide registries include virtually all people living in the country, with one unified public insurance system covering all working people. This makes it possible to undertake these kinds of studies [22].

The aim of this study was to investigate associations between occupational groups defined by sector and psychosocial working conditions, and DP due to MSD, while accounting for familial factors. We hypothesized that there would be significant differences in the magnitude and direction of the associations of different occupational groups defined by sector and psychosocial working conditions, with future DP due to MSD.

## Methods

The method used was a prospective cohort study of twins.

### Study population and data

Twins were identified from the Swedish Twin Register [23], which provided information on date of birth, sex, pair identification, and zygosity and included all individuals who were eligible to be enrolled in the prospective Swedish twin cohort study of disability pension and sickness absence (STODS) [24]. The study sample (N=42 715) included all twins born between 1928 and 1958 in Sweden, who on 1 January 1993 were alive, living in Sweden, below the age of 65; not on DP or old-age pension in December 1992, and were registered as working in 1990 according to the Census Register of Statistics Sweden. The individuals were followed up from 1 January 1993 until DP, old-age pension, 65 years of age, death, or end of follow-up (31 December 2008). Opposite-sex twins and twins without known zygosity were excluded. Data were available for 10 225 same-sex twin pairs with known zygosity (4154 monozygotic [MZ], 6071 dizygotic [DZ]), and for 4093 individuals without their co-twins. Hence the final sample included 24 543 individuals. In 1990, their mean age was 45, at a range of 32–62 years. Information on occupation, educational level, marital status, children living at home, and type of living area in 1990 was obtained from Statistics Sweden. Date of death was obtained from the Causes of Death Register, maintained by the National Board of Health and Welfare.

### Disability pension

Data on DP, including date of being granted DP and DP diagnosis based on the International Classification of Diseases (ICD) were obtained from the Swedish National Social Insurance Agency. The focus was on DP due to MSD (ICD-10 codes M00-M99). All people living in Sweden are covered by the same social insurance system. Any individual above the age of 16 can be granted DP if a disease or injury permanently reduces their work capacity. The amount of DP can be either a minimum benefit, or up to 65% of lost income. Old-age pension can usually be taken at the age of 65.

### Occupational groups

The 1985 version of the Nordic Standard of Occupational Classification (NYK) was used for coding occupations at the three digit level (320 codes). For statistical analyses, the NYK codes were categorized into eight occupational groups defined by sector: administration& management [used as the reference group, NYK codes 200–299]; technology, science, social science& art (NYK codes 000–099); health care and social work (NYK codes 100–199); commercial work (NYK codes 300–399); agriculture forestry& fishing (NYK codes 400–499); transport (NYK codes 600–699); production& mining (NYK codes 500–599 and 700–899); and service& military work (NYK codes 900–989).

### Psychosocial working conditions

The occupational codes were used to measure psychosocial working conditions as proposed in the Job-Demand-Control-Support model [25] via the psychosocial Job Exposure Matrix (JEM) [26]. The JEM is based on data from the 1989–1997 Swedish Work Environment Surveys, covering approximately 50 000 individuals. In the JEM, items measuring the dimensions of job demands (5 items), job control (7 items), and social support at work (4 items) were identified by the factor analyses of all the items included in the survey [26]. The JEM also included occupational code, sex, and age group of the same occupation. The JEM has shown acceptable validity in correlations matching its scores with self-reported equivalents for separate working conditions and by comparing attributed and self-reported scores on back pain, sleep disturbance and mortality. Such agreements were found to be moderate for job control and somewhat lower for job demands and social support [26]. Based on the JEM, each cohort individual was assigned an age-, sex-, and occupation-specific mean score (range 1–10) for job demands, job control (i.e. decision authority and skill discretion), and social support. The higher scores indicated more favourable characteristics: low demands, high control and support. Accordingly, lower scores indicated the opposite. In the analyses, the scores for job demands, job control, and social support were used as continuous variables.

Furthermore, for all the individuals, the psychosocial working condition scores were used to create a “job strain” score. Job demands (median [md] 6.13), job control (md 6.79), and social support (md 6.33) were divided by the median into high and low scores [6]. The high (above median) and low (median value and below) scores were then used to create combinations of scores based on a 2 × 2 table. The job strain scores were high job strain (combination of high demands = below median, low control=below median), low job strain (low demands = above median, high control = above median), active job (high demands =below median, high control = above median), passive job (low demands =above median, low control = below median). In addition to the above categories, a fifth category was created by adding the dimension of low social support to high strain: iso strain (high demands =below median, low control = below median, low support = below median).

### Covariates

Age was used as a continuous variable in the analyses, but for descriptive purposes we categorized age into three categories: 32–44, 45–54, and 55–62 years. Men were used as the reference group for sex. Education was years of education (continuous) in the statistical analyses, and for descriptive purposes categorized as: low (≤ 9 years of education), intermediate (10–12 years of education), and high (≥ 13 years of education). Marital status was dichotomized into married (this served as the reference group) and unmarried (including single, divorced, widow/widower). Having children living at home was dichotomized into no children living at home versus at least one child living at home (reference category). For type of living area: urban/semi-urban areas (reference group) were compared with semirural/rural areas.

### Statistical analyses

**Figure 1:**
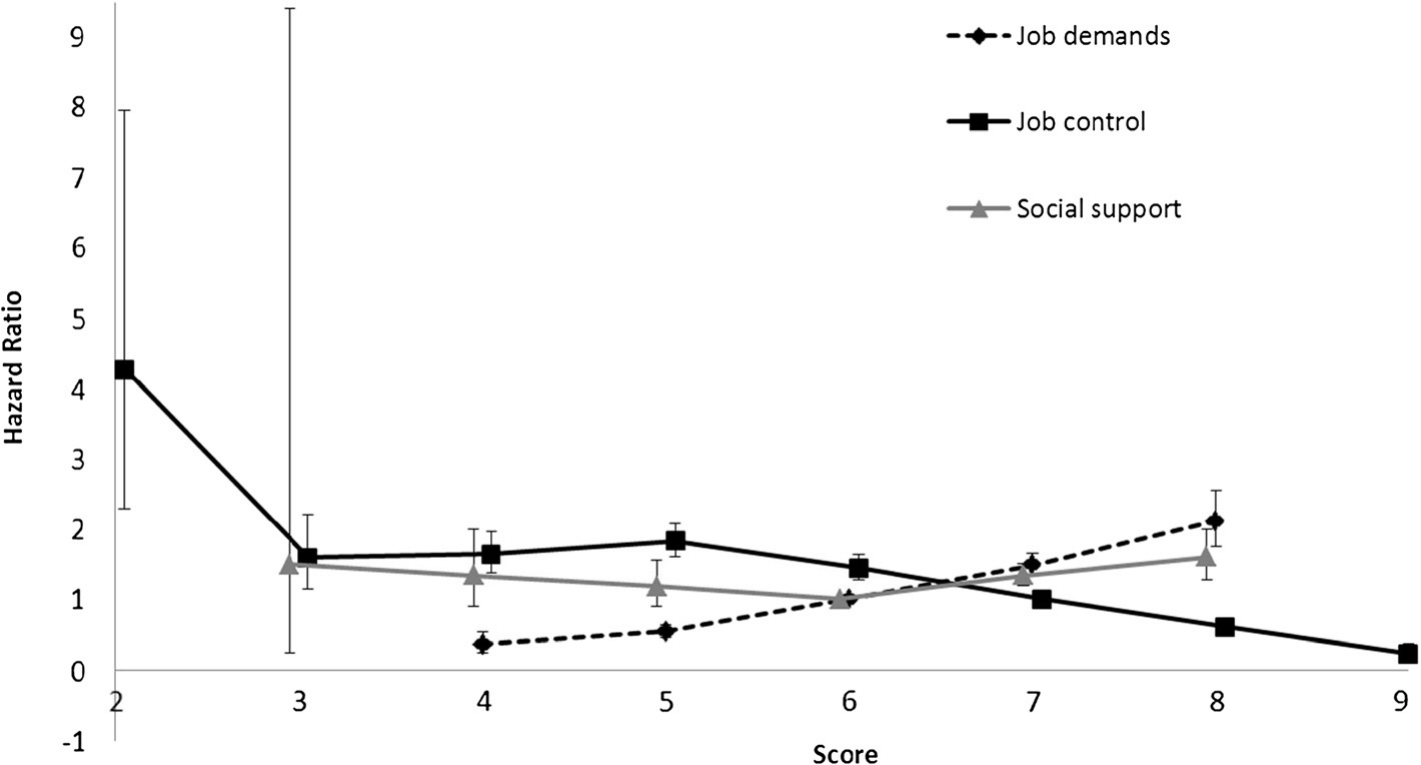
**Hazard ratios for each value of psychosocial working conditions.** Error bars indicate 95% confidence intervals.

Cox proportional hazards regression models were first calculated for the whole twin cohort and then for complete twin pairs discordant for the outcome (co-twin control). Hazard ratios (HR) with 95% confidence intervals (CI) were computed, using follow-up time in days, with DP due to MSD as the outcome variable. The reference group was those with no DP or DP due to another reason. There was no major influence on HRs, regardless of whether or not the reference group included those with DP due to any other reason. The proportional hazards assumption was graphically tested by observing that the ‘log-log’ curves for the categories of risk factors were parallel and also tested by Schoenfeld residuals with no violations detected. All analyses were age adjusted and clustered on pair identity to adjust 95% CIs for any possible intra-pair correlations. Next we analysed the data separately for women and men, and found no major differences in the hazard estimates between the sexes. Therefore, we decided to combine women and men in all analyses and adjusted all analyses for sex. In order to assess the linear trend in psychosocial working conditions, we calculated HR with 95% CI for each value within a risk factor (Figure 1). The lowest and highest values of psychosocial working conditions were excluded from the figure due to their low frequencies, leaving values of 4–8 for job demands, 2–10 for job control and 3–8 for social support. The reference group for the linear trend evaluation was set to the modal value, this being 6 for job demands and social support, and 7 for job control.

In order to investigate the independent effect of sector-defined occupational group, psychosocial working conditions and job strain, we further adjusted the models for the potential confounders found to be significant in the separate analyses, including children living at home, marital status, educational level and type of living area. Furthermore, to investigate the potential effect of occupational group on the associations between psychosocial working conditions and DP due to MSD, and the effect of psychosocial working conditions on the associations between sector-defined occupational group and DP due to MSD , we ran the analyses by including occupational group as an additional covariate for psychosocial working conditions and vice versa.

Next, the analyses were conducted using twin pairs discordant for DP; i.e. one twin actually received DP due to MSD while the other twin had not been granted DP due to MSD during the follow-up time. In these conditional Cox proportional hazards regression models, the follow-up time to DP of one twin individual within a twin pair, in relation to the follow-up time of the co-twin, was analysed with stratification for twin pair identity, allowing each twin pair their own baseline hazard. Stratification made it possible to control for the effects of potentially confounding familial factors. In a twin sample whose twin pairs have been reared together and share both genetic make-up and home and family environment, several familial factors are present. Therefore, the actual associations found between risk factors and DP would not be explained by familial factors shared by the twin pair. In twin data, if the association between risk factors and DP is because of family background, the association should be found only between, but not within, twin pairs reared together, meaning that the association should be found in the analyses of the whole cohort but not in discordant twin pairs. Second, if genetic factors and family background play a role, the association should be present within DZ twin pairs, who share on average 50% of their segregating genes, but not within MZ twin pairs, who share 100% of genes. Furthermore, if the association is independent of familial effects (caused by non-familial environmental factors) it should be found in both MZ and DZ twin pairs. The statistical analyses were performed using Stata version 9.2 (Stata Corporation, College Station, TX, USA).

The study was approved by the Regional Ethical Review Board in Stockholm (2007/524-31).

## Results

The mean follow-up time was 12 years (SD 4.3) and 7% of the final sample was granted DP due to MSD during the follow-up (Table 1). The age- and sex-adjusted HRs revealed that being female, unmarried, having no children, living in a semi-rural or rural area and, compared to the administration and management sector, work in health care and social work, agriculture, forestry and fishing, transportation, production and mining, and service and military work sectors were each associated with a two- to three-fold increased likelihood of receiving DP due to MSD, whereas each single year of more education predicted lesser risk (Table 2). Furthermore, each single unit decrease in job demands and each single unit increase in social support predicted an increased risk, whereas each single unit increase in job control decreased the risk of DP in the age- and sex-adjusted model (Table 2). Figure 1 shows the HR with 95% CI for each value of job demand, job control and social support. The combination score of Job-Demand-Control-Support (job strain) indicated that having a high strain or active job predicted a lower risk of DP due to MSD, whereas having a passive job predicted a significantly higher risk.

**Table 1:** Frequencies (percentages) and means (standard deviations) of background factors, occupational groups, psychosocial working conditions, and job strain in 1990 for those granted DP due to musculoskeletal disorders (MSD), those granted DP due to some other reason (DP other) and those not receiving DP (No DP) during 1993–2008 follow-up (n=24 543)

	**DP MSD n= 1 774 (7%)**	**DP other n= 2 480 (10%)**	**No DP n= 20 290 (83%)**
**Sex**	**n (%)**	**n (%)**	**n (%)**
Men	655 (37)	1 045 (43)	10 445 (51)
Women	1 119 (63)	1 426 (58)	9 845 (49)
**Zygosity**			
MZ	678 (38)	969 (39)	8 058 (40)
DZ same-sex	1 096 (62)	1 511 (61)	12 232 (60)
**Age groups**			
32–44	638 (36)	1 056 (43)	10 827 (53)
45–54	937 (53)	1 123 (45)	6 401 (32)
55–62	199 (11)	301 (12)	3 062 (15)
**Marital status**			
Married	1 191 (67)	1 510 (61)	13 801 (68)
Unmarried	583 (33)	970 (39)	6 489 (32)
**Children living at home**			
Yes	1 022 (58)	1 399 (56)	12 936 (64)
No	752 (42)	1 081 (44)	7 354 (36)
**Educational level**			
High (≥13 years)	161 (9)	527 (21)	5 319 (26)
Intermediate (10–12 years)	772 (44)	1 051 (42)	8 573 (42)
Low (≤9 years)	841 (47)	902 (36)	6 398 (32)
**Type of living area**			
Urban/semi-urban	1 097 (62)	1 708 (69)	13 994 (69)
Semirural/rural	677 (38)	772 (31)	6 296 (31)
**Occupational groups defined by sector**			
Administration& management	172 (10)	362 (15)	3 514 (17)
Technology, science, social science& art	143 (8)	391 (16)	3 819 (19)
Health care& social work	395 (22)	513 (21)	3 451 (17)
Commercial work	107 (6)	157 (6)	1 683 (8)
Agriculture, forestry& fishing	60 (3)	75 (3)	718 (4)
Transport	111 (6)	140 (6)	1 045 (5)
Production& mining	500 (28)	560 (23)	4 143 (20)
Service& military work	286 (16)	282 (11)	1 917 (9)
**Job strain**			
High strain	217 (12)	344 (14)	2 784 (14)
Low strain	101 (6)	177 (7)	1 631 (8)
Active	370 (21)	774 (31)	7 995 (39)
Passive	926 (52)	954 (38)	6 336 (31)
Iso-strain	160 (9)	231 (9)	1 544 (8)
**Psychosocial working conditions**	**mean (SD)**	**mean (SD)**	**mean (SD)**
Job demands (range 1–10, high score is low)	6.34 (0.74)	6.12 (0.79)	5.99 (0.76)
Job control (range 1–10, high score is high)	6.04 (1.25)	6.42 (1.28)	6.67 (1.27)
Social support (range 1–10, high score is high)	6.46 (0.66)	6.40 (0.62)	6.34 (0.63)

**Table 2:** Cox proportional hazard ratios (HR) with 95% confidence intervals (CI) of background factors, occupational groups, psychosocial working conditions and job strain for being granted DP due to MSD during the follow-up

	** Age- and sex-adjusted**^**1**^			** Discordant twin pairs**^**2**^		
			** MZ (n = 427 pairs)**		** DZ (n = 745 pairs)**	
	**HR**	**95% CI**	**HR**	**95% CI**	**HR**	**95% CI**
**Sex** (being a woman)	**1.73**	**1.57, 1.91**	-	-	-	-
**Marital status** (unmarried)	**1.15**	**1.05, 1.27**	1.01	0.83, 1.24	1.07	0.91, 1.25
**Children living at home** (no)	**1.32**	**1.19, 1.45**	0.97	0.79, 1.21	**1.41**	**1.20, 1.67**
**Education** (number of years)	**0.82**	**0.80, 0.84**	0.94	0.88, 1.01	**0.84**	**0.81, 0.88**
**Type of living area** (semi-rural/rural)	**1.34**	**1.21, 1.47**	1.00	0.75, 1.33	**1.41**	**1.12, 1.76**
**Occupational groups defined by sector**						
Administration& management	1.00	referent	1.00	referent	1.00	referent
Technology, science, social science& art	0.95	0.76, 1.18	1.27	0.76, 2.13	1.33	0.93, 1.88
Health care& social work	**2.06**	**1.72, 2.46**	**1.61**	**1.05, 2.48**	**1.94**	**1.44, 2.66**
Commercial work	**1.54**	**1.21, 1.95**	1.03	0.62, 1.72	**1.46**	**1.01, 2.10**
Agriculture, forestry& fishing	**2.33**	**1.74, 3.14**	2.01	0.94, 4.30	**2.37**	**1.41, 3.98**
Transport	**2.88**	**2.27, 3.66**	**2.68**	**1.35, 5.33**	**3.16**	**2.04, 4.89**
Production& mining	**3.51**	**2.92, 4.21**	**1.88**	**1.15, 3.08**	**2.83**	**2.03, 3.94**
Service& military work	**2.90**	**2.40, 3.51**	1.48	0.91, 2.43	**2.71**	**1.94, 3.78**
**Psychosocial working conditions**						
Job demands (range 1–10, high score is low)	**1.63**	**1.54, 1.73**	**1.17**	**1.00, 1.37**	**1.35**	**1.20, 1.53**
Job control (range 1–10, high score is high)	**0.76**	**0.73, 0.78**	**0.80**	**0.72, 0.89**	**0.79**	**0.74, 0.85**
Social support (range 1–10, high score is high)	**1.17**	**1.05, 1.30**	0.90	0.75, 1.09	0.94	0.81, 1.10
**Job strain**						
High strain	**0.75**	**0.59, 0.96**	**1.87**	**1.07, 3.29**	0.81	0.55, 1.21
Low strain	1.00	referent	1.00	referent	1.00	referent
Active	**0.60**	**0.51, 0.71**	1.06	0.76, 1.50	0.79	0.60, 1.03
Passive	**1.54**	**1.33, 1.80**	**1.41**	**1.03, 1.94**	**1.35**	**1.04, 1.75**
Iso-strain	1.15	0.93, 1.41	**2.84**	**1.58, 5.11**	**1.43**	**1.00, 2.03**

Notes: ^**1**^ Clustered on pair identity, ^**2**^MZ=monozygotic, DZ=dizygotic.

When accounting for familial confounding (Table 2), the predictive values of most of the occupational groups were clearly confirmed to be independent of any familial influence. For a few of the sector-defined occupational groups (such as technology, science, social science& art, and commercial work), the point estimates did change slightly, but the CIs remained overlapping with the analyses from the whole cohort. In addition, most of the associations between psychosocial working conditions and DP remained, i.e. each single unit decrease in job demands predicted an increased risk, and each single unit increase in job control predicted a lower risk of DP. The job strain evaluation revealed that a high strain or passive job remained associated with an increased risk of DP, while an iso-strain job became a significant predictor of increased risk. All other psychosocial working conditions revealed HR values of around the same magnitude and direction in the discordant pair analyses as those in the analysis of the whole cohort.

Furthermore, the models with additional confounders were fitted to account for the potential effect of marital status, children living at home, area of living, and education in the association between sector-defined occupational groups, psychosocial working conditions and DP due to MSD. These additional confounders had no major influence, as shown in the comparison between the point estimates in the age- and sex-adjusted model Table 2 and those in Models 1 and 2 in Table 3.

**Table 3:** Cox proportional hazard ratios with 95% confidence intervals (CI) of occupational groups, psychosocial working conditions and job strain for being granted DP due to MSD during the follow-up

	** Model 1**		** Model 2**	
	**HR**	**95% CI**	**HR**	**95% CI**
**Occupational groups defined by sector**				
Administration& management	1.00	referent	1.00	referent
Technology, science, social science& art	**1.27**	**1.01, 1.59**	**1.27**	**1.01, 1.60**
Health care& social work	**2.13**	**1.78, 2.55**	**2.19**	**1.81, 2.65**
Commercial work	**1.28**	**1.01, 1.63**	1.20	0.94, 1.53
Agriculture, forestry& fishing	**1.89**	**1.40, 2.54**	**1.49**	**1.07, 2.05**
Transport	**2.35**	**1.84, 3.00**	**1.89**	**1.40, 2.55**
Production& mining	**2.63**	**2.18, 3.18**	**2.16**	**1.72, 2.72**
Service& military work	**2.37**	**1.95, 2.87**	**1.96**	**1.56, 2.46**
**Psychosocial working conditions**				
Job demands (range 1–10, high score is low)	**1.31**	**1.22, 1.41**	**1.10**	**1.02, 1.20**
Job control (range 1–10, high score is high)	**0.85**	**0.82, 0.88**	**0.93**	**0.89, 0.97**
Social support (range 1–10, high score is high)	1.05	0.95, 1.15	1.08	0.98, 1.19
**Job strain**				
High strain	0.88	0.69, 1.12	0.93	0.73, 1.19
Low strain	1.00	referent	1.00	referent
Active	**0.76**	**0.64, 0.90**	0.89	0.75, 1.07
Passive	**1.33**	**1.14, 1.55**	**1.25**	**1.07, 1.46**
Iso-strain	**1.32**	**1.08, 1.63**	**1.27**	**1.04, 1.57**

Model 1: Additionally (after taking age, sex and pair-cluster into account) adjusted for marital status, children living at home, area of living, and years of education.

Model 2: As Model 1, but additionally adjusted for occupational groups, or psychosocial working conditions.

## Discussion

Our population-based twin cohort sample of 24 543 twin individuals with complete register data on occupational groups defined by sector, psychosocial working conditions and several confounding factors was used to explore the factors associated with DP due to MSD during an average of 12 years’ follow-up. To the best of our knowledge, this is the first study to use register data with which it is possible to control for familial confounding in sector-defined occupational groups and psychosocial working conditions for DP due to MSD. This unique dataset revealed that several sector-defined occupational groups and also job strain (such as passive jobs) have a direct association with DP due to MSD, i.e., they are not dependent on familial confounding in terms of genetics and shared environment. Furthermore, occupational groups and psychosocial working conditions were mainly independent predictors when several confounding factors from the same time point were taken into account, namely marital status, children living at home, area of living, and years of education.

Another main finding of this study was the direct association of psychosocial working conditions with DP due to MSD. To date, very few studies have investigated the associations of psychosocial working conditions or their combinations with DP due to MSD. The few existing studies have indicated that low job control [9,10] or high job strain [11-14] predict a higher risk of DP in general, and are thus in line with our findings for DP due to MSD. The effect of job demands, job control, social support, high strain, passive job, and iso-strain on the risk of DP due to MSD was mainly independent of familial effects, suggesting that interventions at workplaces would be potentially beneficial. These could include measures at the organizational level to reduce or prevent disability due to MSD. Furthermore, a recent study showed that psychosocial working conditions have only a moderate effect on the risk of DP but that they nevertheless confound the associations between occupations and DP [5]. Our results support the finding that psychosocial working conditions have a confounding effect, based on the small but decisive changes in the effects of occupational groups on the risk of DP when taking into account psychosocial working conditions. However, in our data, these psychosocial working conditions seemed to relatively strongly and independently (i.e. they were not dependent on confounding factors and family background) predict DP due to MSD. This may reflect the fact that the pathway to DP might not be the same for all DP diagnoses [27], since previous studies of psychosocial working conditions have investigated the risk of DP in general without reference to specific diagnosis groups. Hence, in further studies, the diagnosis leading to DP should be taken into account when investigating risk factors.

However, these associations do seem to have some complexity. For example, with respect to sector-defined occupational groups, not all the associations with DP were fully confirmed when familial confounding was taken into account. The point estimates changed somewhat in discordant pair analyses, although the magnitude remained around the same and the change in the direction of the estimates was rather small. This suggests that an even larger sample size with more precisely determined occupational groups would be needed to clarify these associations and the possible influencing factors. Nonetheless, our results indicate that these associations were mainly independent of familial confounding; but future research will be needed to examine some characteristics of these occupational groups or psychosocial working conditions to explain why their association with DP could be influenced by familial effects (including genetics and shared environment such as social background and values within families). This twin study is, to the best of our knowledge, the first to study familial factors in the association between sector-defined occupation groups, psychosocial working conditions and future DP due to MSD. It is based on the knowledge that genetics play a role in DP, irrespective of the age when DP was granted or the DP diagnosis group [16,17]. Another mechanism linking family background to DP is the fact that many risk factors (including medical disorders and other factors such as education and income, see [28,29]), have a genetic component and may therefore affect associations between risk factors and DP. In addition, circumstances during childhood, for instance, health behaviours may be established early within the family, and ways in which to handle disease and functional disabilities (for example by being absent due to sickness) can be passed on from parents to children, further indicating the influence of family background.

The results from many other studies of work-related factors such as occupational socioeconomic status, physical work loading, and psychosocial aspects [3,7,8] and how they predict DP due to MSD are in line with our results. However, these previous studies have only had access to self-reported data of work-related factors [7,8,10,18] thus being potentially subject to reporting bias, for example, as they are influenced by work capacity and non-reporting, etc. Bias in such studies may also be due to dropouts. Here, we were able to avoid reporting bias, since only register data were used. However, one of the limitations of this study is partially related to this kind of register data. The coding of occupational groups was performed according to the NYK system, which groups similar occupations into sector-defined groups without taking into account industrial sectors, employment status (such as being an entrepreneur or employed), variations due to different work organization, or years of education. Therefore, these sector-defined occupational groups are somewhat overlapping and may include both high and low levels of risks, based on social composition and psychosocial working conditions [15].

Another limitation of this study is also potentially related to the register data, i.e. these kinds of studies are only available in countries with a high coverage of national registries and permission to link data between registries. Therefore, these results may be applicable to other Nordic countries that have similar social systems and schemes, but perhaps less so to other countries. The strengths of this study are the longitudinal cohort design, the large sample size, the 15-year prospective follow-up from 1993 to 2008, and the fact that there were no losses in the follow up. Most of the published studies on work-related factors and the risk of DP due to MSD have had shorter follow-up times; 5–12 years [7,10,13,18] except for one Finnish study with a 30-year follow-up [8]. Most investigations have been based on certain occupational groups [4] or distinct industrial sectors [7,10] attempting to identify work-related risk factors for DP with MSD. One must speculate on the extent to which their results are due to selection effects of different jobs or working conditions. A further strength of our study is the use of the co-twin control design, which provides an elegant way to control for familial confounding. Through this design it was possible to analyse twins who were discordant for both DP and work-related factors; this represent a powerful tool, as it allows cases and controls to be matched optimally being twins. The comparison of MZ and DZ discordant twins revealed the trivial role of family background and genetic factors with regard to the influence of most occupational groups and psychosocial working conditions such as job strain on DP. These proved to be direct risk factors for DP due to MSD.

## Conclusions

Occupational groups and psychosocial working conditions seem to be risk factors for DP due to MSD and are independent of familial confounding. These associations are also independent of other socioeconomic factors that potentially influence the pathway to DP.

## Competing interests

The authors declare that they have no competing interests.

## Authors’ contributions

AR, KA, and PS contributed to the conception and design of the study, PS to the acquisition of data, and all the authors to the analysis and interpretation of data. AR and PS drafted the article and all the authors revised it critically as regards important intellectual content. All the authors have given their final approval for the manuscript to be submitted.

## Pre-publication history

The pre-publication history for this paper can be accessed here:

http://www.biomedcentral.com/1471-2474/14/268/prepub
